# Effect of intracerebroventricular epinephrine microinjection on blood pressure and urinary sodium handling in gestational protein-restricted male adult rat offspring

**DOI:** 10.1242/bio.038562

**Published:** 2019-04-15

**Authors:** Bárbara Vaccari Cardoso, Augusto Henrique Custódio, Patrícia Aline Boer, José Antonio Rocha Gontijo

**Affiliations:** Fetal Programming Laboratory and Hydroelectrolyte Metabolism Laboratory, Nucleus of Medicine and Experimental Surgery, Department of Internal Medicine, Faculty of Medical Sciences at State University of Campinas, Campinas 13083-894, São Paulo, Brazil

**Keywords:** Fetal programming, Central nervous system, Renal function, Maternal protein-restriction, Natriuresis, Arterial hypertension, Adrenergic system

## Abstract

In this study, we hypothesized that blunting of the natriuresis response to intracerebroventricularly (ICV) microinjected adrenergic agonists is involved in the development of hypertension in maternal low-protein intake (LP) offspring. A stainless steel cannula was stereotaxically implanted into the right lateral ventricle (LV), then we evaluated the ICV administration of adrenergic agonists at increasing concentrations, and of α1 and α2-adrenoceptor antagonists on blood pressure and urinary sodium handling in LP offspring relative to an age-matched normal-protein intake (NP) group. We confirmed that epinephrine (Epi) microinjected into the LV of conscious NP rats leads to enhanced natriuresis followed by a reduction in arterial pressure. This response is associated with increased proximal and post-proximal sodium excretion accompanied by an unchanged glomerular filtration rate. The current study showed, in both NP and LP offspring, that the natriuretic effect of Epi injection into the LV was abolished by prior local microinjection of an α1-adrenoceptor antagonist (prazosin). Conversely, LV α2-adrenoceptor antagonist (yohimbine) administration potentiated the action of Epi. The LV yohimbine pretreatment normalized urinary sodium excretion and reduced the blood pressure in LP compared with age-matched NP offspring. These are, as far as we are aware, the first results showing the role of central adrenergic receptors’ interaction on hypertension pathogenesis in maternal LP fetal-programming offspring. This study also provides good evidence for the existence of central nervous system adrenergic mechanisms consisting of α1 and α2-adrenoceptors, which work reciprocally on the control of renal sodium excretion and blood pressure. Although the precise mechanism of the different natriuretic response of NP and LP rats is still uncertain, these results lead us to speculate that inappropriate neural adrenergic pathways might have significant effects on tubule sodium transport, resulting in the inability of the kidneys to control hydrosaline balance and, consequently, an increase in blood pressure.

## Abstract (precis)

**Summary:** We evaluated the effect of intracerebroventricular microinjections of adrenergic agonists at increasing concentrations, and μ1 and μ2-adrenergic receptors antagonists on blood pressure and urinary sodium in hypertensive low- and normal-protein rat offdpring.

## INTRODUCTION

Environmental and genetic factors influence ontogenetic development, and in adverse circumstances eventually lead to functional and structural disorders in tissues and organs. In rats, gestational protein restriction is associated with low birthweight, fewer nephrons and increased risk of development of heart disease, kidney dysfunction and metabolic syndrome in adult life (Ashton, 2000; Barker, 1998; Mesquita et al., 2010a,b; Dasinger et al., 2016; Vaccari et al., 2015; Sene et al., 2013). We recently demonstrated that maternal low-protein intake (LP) offspring have a lower birth weight, 30% fewer nephrons and arterial hypertension (Mesquita et al., 2010a,b; Vaccari et al., 2015) when compared with an age-matched, normal-protein intake (NP) group.

Additionally, water and salt balance studies have shown that arterial hypertension in LP offspring is associated with decreased urinary sodium excretion (Mesquita et al., 2010a,b; Dasinger et al., 2016; Vaccari et al., 2015; Sene et al., 2013; Custódio et al., 2017). The involvement of the central nervous system (CNS) in the control of blood pressure and water and salt homeostasis has been demonstrated in several studies (Gontijo and Kopp, 1994; Gontijo et al., 1992; DiBona, 2000; Scabora et al., 2015). It has long been known that there is an association between the CNS and the control of water and salt excretion by the kidneys (Ashton, 2000; Silva-Netto et al., 1985, 1986). Adrenergic stimulation of the septal area, lateral hypothalamus, subfornical organ and the anterior region of the third ventricle induces dose-related natriuresis accompanied by, to a lesser extent, kaliuresis (Gontijo and Kopp, 1994; Dorn et al., 1970; Pillar et al., 1977; Camargo et al., 1976; Saad et al., 1976; Brody et al., 1980; Bealer, 1983; Lutaif et al., 2015). Conversely, studies have shown that electrolytic lesion of the hypothalamic regions in conscious rats reduces salt intake and the pressor response to cholinergic and noradrenergic microinjection into the median preoptic nucleus (Covian et al., 1975). Prior findings have also revealed that central α- and β-adrenergic receptors response are involved in CNS hydrosaline homeostasis (Gontijo and Kopp, 1994; Covian et al., 1975; Silva-Netto et al., 1983, 1980; Franci et al., 1980; Gontijo et al., 1991; Gontijo et al., 1990).

It has been postulated that the kidneys are of crucial importance in the pathogenesis of arterial hypertension as a consequence of primary tubule disorder or renal hemodynamics dysfunction promoting change in tubular sodium and water handling (Johns et al., 2011; Boer et al., 2005; Xu et al., 2014; Hall et al., 2012). Although the precise mechanism underlying the chronic blood pressure increases in maternal LP offspring remains to be elucidated, renal-mediated deregulation mechanisms of fluid and electrolytes balance are believed to dominate long-term control of arterial blood pressure. Experimental studies support the hypothesis that fetal programming is associated with changes in the tubule sodium transporters in different segments of nephron (Manning et al., 2002; Alwasel and Ashton, 2009; Baum, 2010). Research from our laboratory (Custódio et al., 2017) demonstrates that bilateral renal denervation markedly attenuates the increase in arterial pressure and increased tubular sodium excretion in LP offspring. The enhanced urinary sodium excretion in renal denervated LP offspring was accompanied by a significant reduction in proximal tubular sodium reabsorption. This study also demonstrated that impaired dorsal renal ganglia and pelvic neurokinin expression associated with responsiveness of renal sensory receptors are conducive to excess renal reabsorption of sodium and development of hypertension in 16-week-old LP offspring (Custódio et al., 2017). Given these results, we hypothesized that blunting of the natriuresis response to centrally injected epinephrine (Epi) α-agonists/antagonists might affect renal tubule sodium transport, resulting in the inability of the kidneys to handle the hydrosaline balance and consequently, promoting blood pressure enhancement. To test this hypothesis, in this study we evaluated the effect of intracerebroventricular (ICV) microinjection of adrenergic agonists at increasing concentrations, and the effect of α1 and α2 types of adrenoceptor antagonists on blood pressure and urinary sodium handling in LP offspring relative to age-matched normotensive NP counterparts.

## RESULTS

### Dams and offspring parameters

Table 1 shows serum sodium, lithium and potassium levels from NP and LP offspring, with no significant differences in NP rats compared with the LP group. In general, water and food sodium intake, and plasma osmolality were similar in male offspring of NP and LP groups when normalized by body weight (Table 1). Gestational protein restriction did not significantly change the pregnant dams' body mass during gestation (Fig. 2A), nor did it did not affect the number of offspring per litter and the proportion of male and female offspring (*P*=0.3245). The birthweight of LP male pups (*n*=26) was significantly reduced compared with NP pups (*n*=27) (6.238±0.2288 g versus 7.200±0.05163 g; *P*=0.0001), respectively (Fig. 2B). The body mass of 12 days-weaned LP pups (21±0.5 g, *n*=18) remained lower than age-matched NP pups (24±0.6 g, *n*=19) (*P*=0.0017) (Table 1). The body mass of LP pups (67.9±0.99 g, *n*=17) remained lower than age-matched NP pups (72.10±1.42 g, *n*=20) until weaning at 21 days after birth (*P*=0.05) (Fig. 2C). However, between 4 and 16 weeks of age, the body weight of LP and NP rats was not significantly different (*P*=0.5170) (Fig. 2C).

**Table 1.**
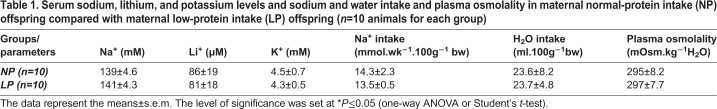
**Serum sodium, lithium, and potassium levels and sodium and water intake and plasma osmolality in maternal normal-protein intake (NP) offspring compared with maternal low-protein intake (LP) offspring (*n*=10 animals for each group)**

**Fig. 1.**
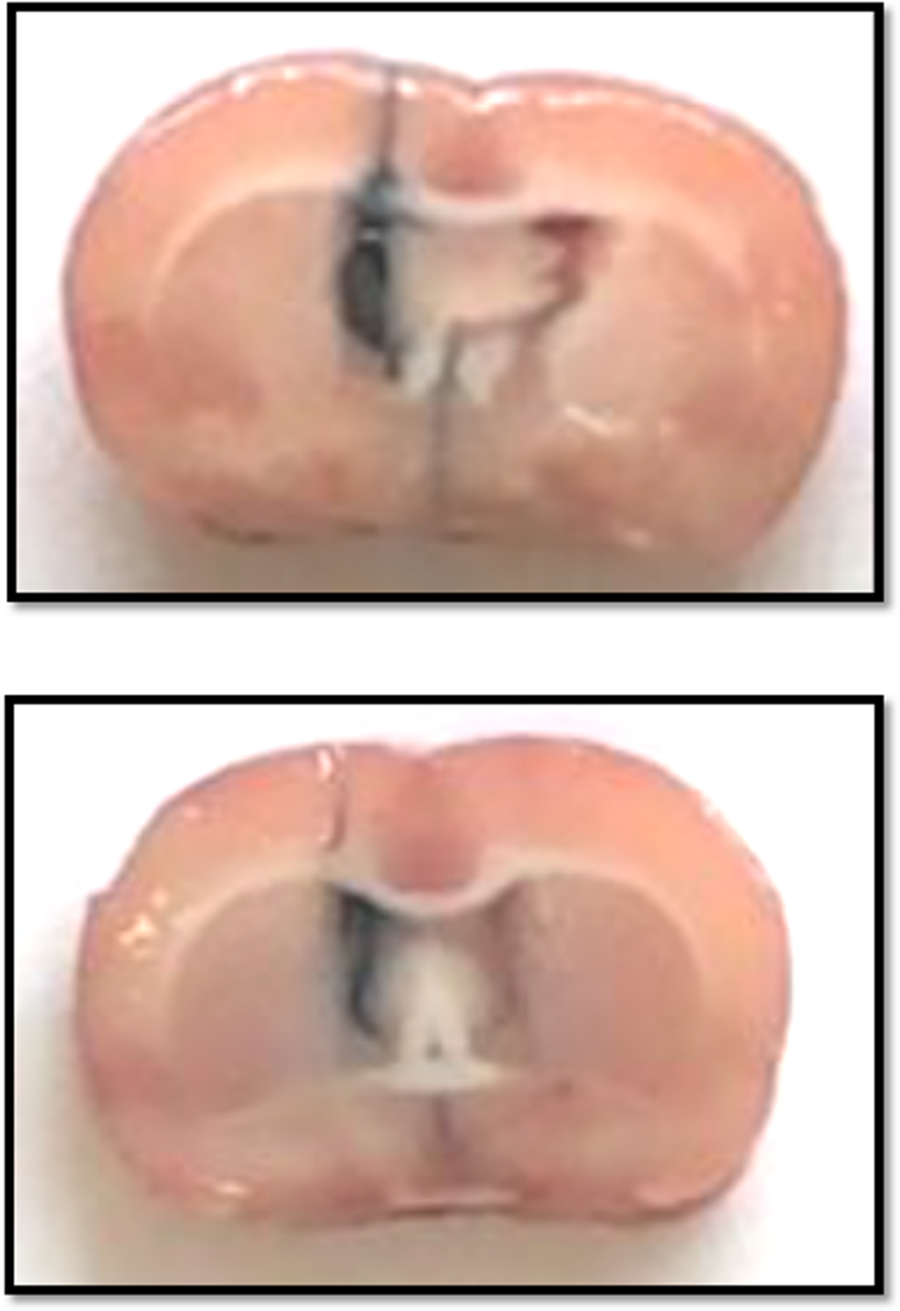
**Histological verification of the cerebral right lateral ventricle (LV) guide cannula.**

**Fig. 2.**
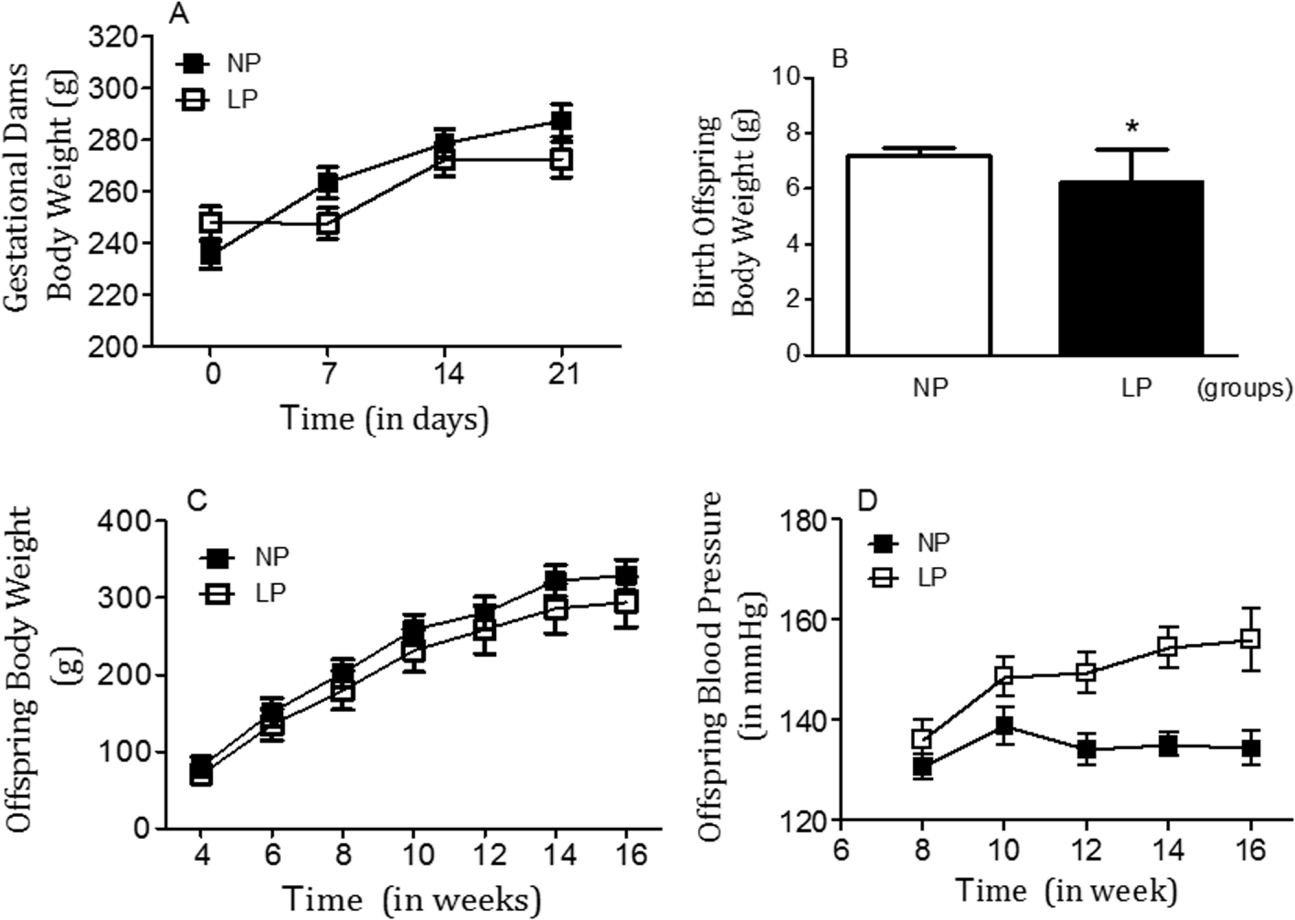
Gestational dams' body weights (A), offspring body weight at birth (B), body weights from 4-day to 16-week-old (C, in grams) and offspring blood pressure from 8 to 16-week-old (in mmHg) NP compared to age-matched LP offspring (D). The results are expressed as means±s.d. Data were analyzed using a two-way ANOVA test with *post-hoc* comparisons by Bonferroni's contrast test. The level of significance was set at *P<0.05.

### Blood pressure measurement

**Fig. 3.**
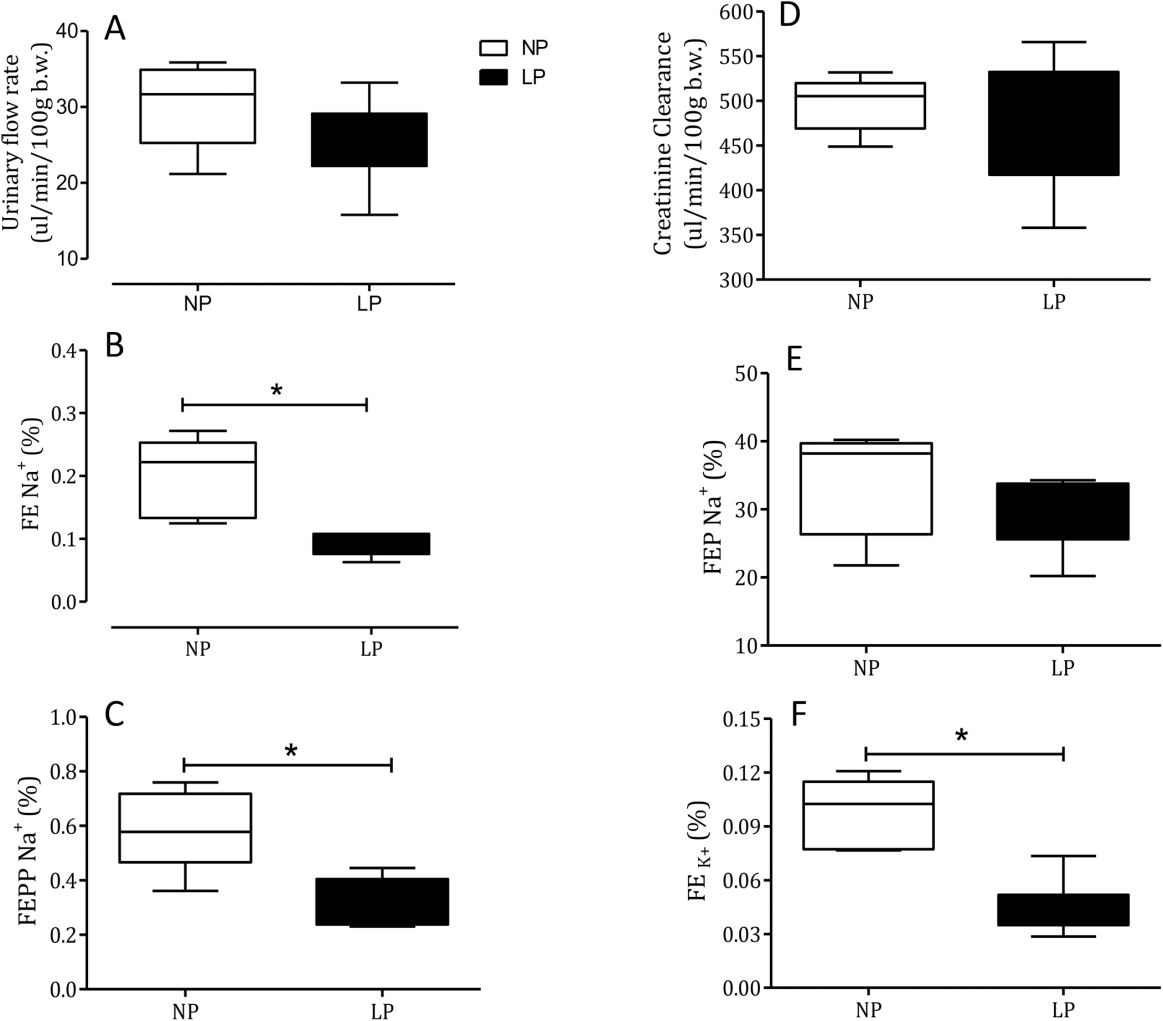
Renal function studies using urinary flow rate (A), fractional sodium excretion (FE*_Na_*+; B), fractional post-proximal sodium excretion (FEPP*_Na_*+; C), fractional proximal sodium excretion (FEP*_Na_*+; E), creatinine clearance (*C_Cr_*; D) and fractional potassium excretion (FE_K_+; F), in male 16-week-old NP compared to age-matched LP (*n*=10 for each group). Results are expressed as median and quartile deviation. Data were analyzed using a one-way ANOVA test. The level of significance was set at *P<0.05.

**Fig. 4.**
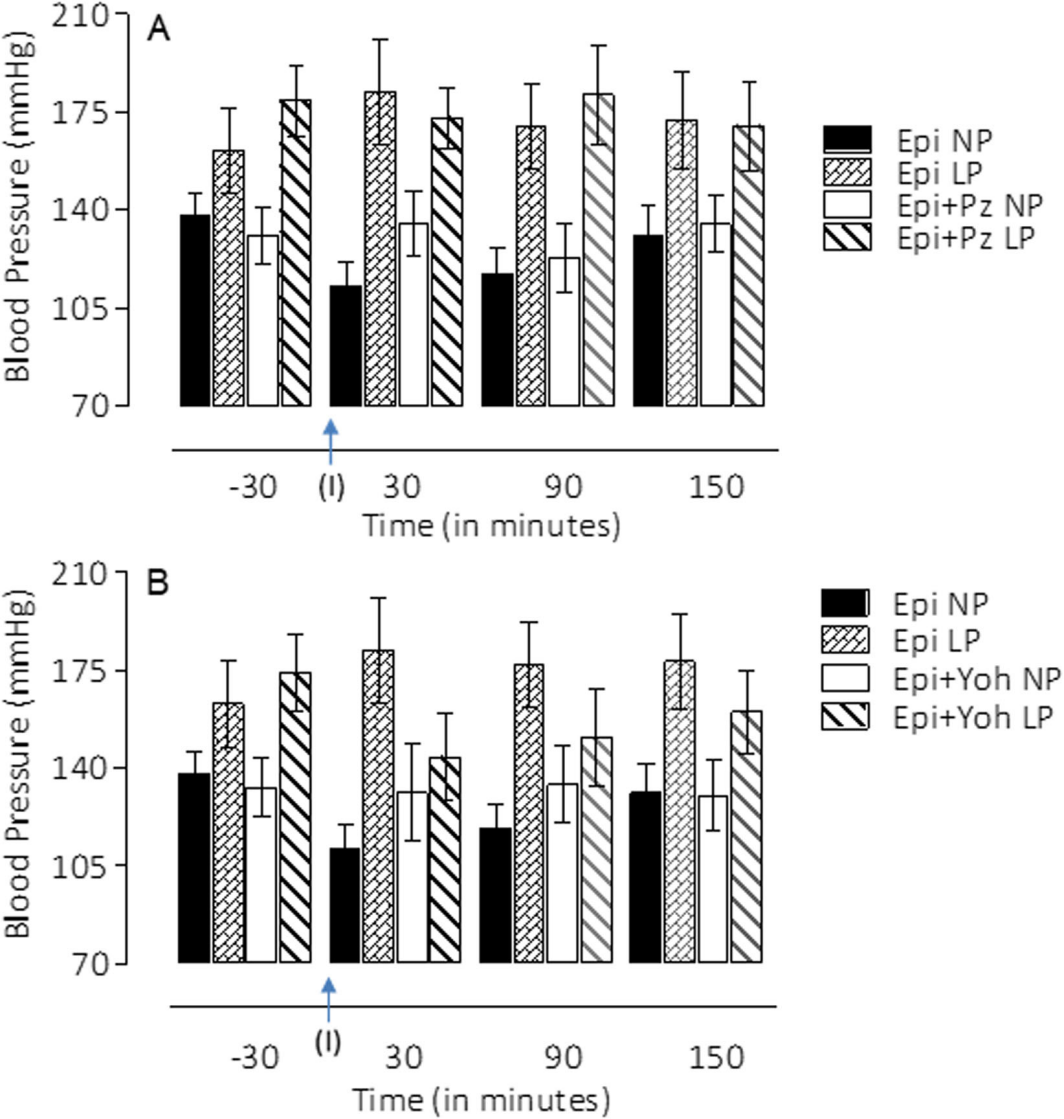
Effect of the LV microinjection (I) of agonist 0.3-µmol Epi, and their antagonists, 4-nmol prazosin (A) and 4 nmol yohimbine (B), on blood pressure among NP group relative to LP. Data were analyzed using a two-way ANOVA test with *post-hoc* comparisons by Bonferroni's contrast test. The level of significance was set at *P<0.05.

**Fig. 5.**
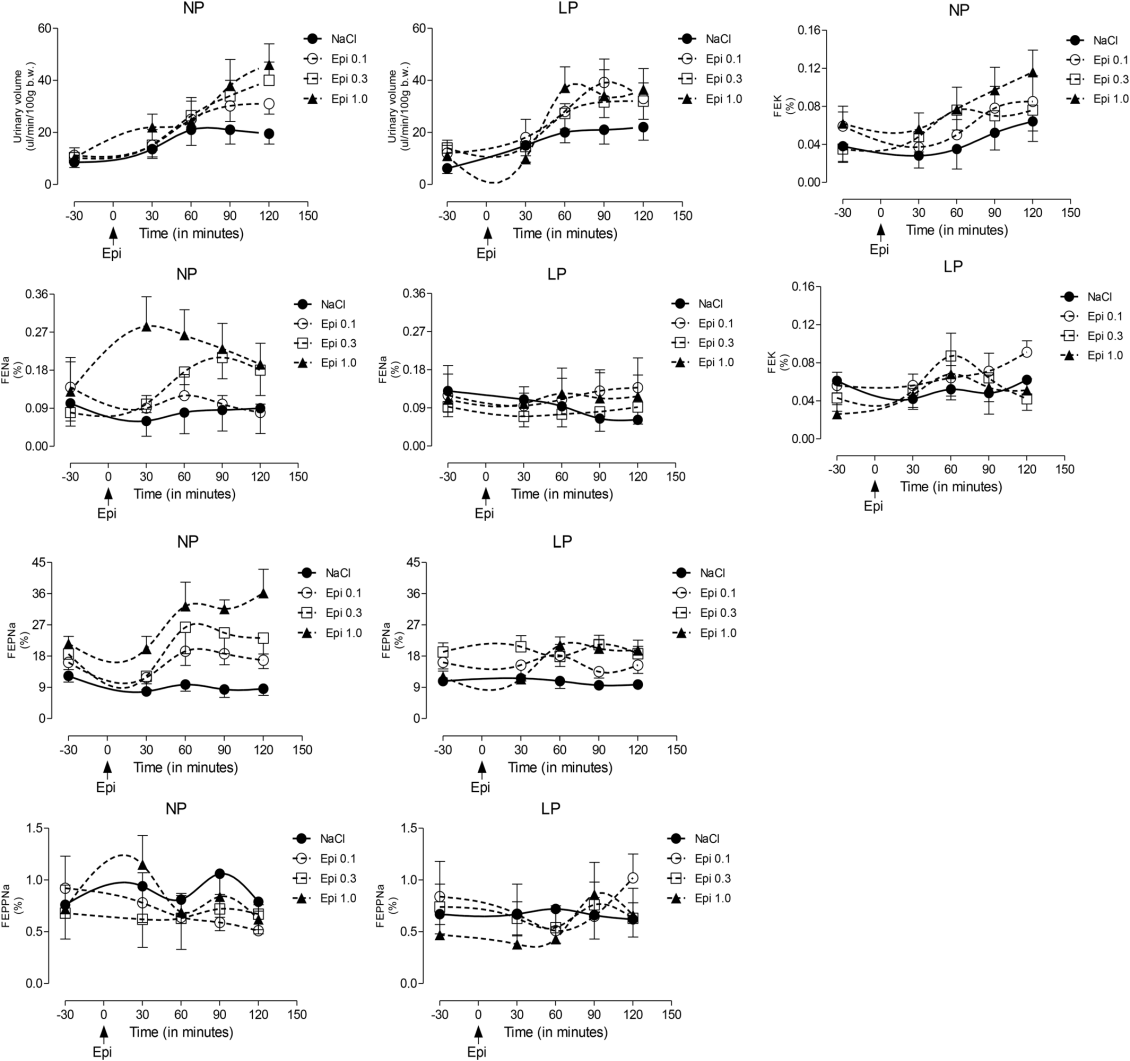
Dose-response curve to LV microinjection of agonists at different concentrations of Epi (0.1, 0.3 and 1.0 µmol) in a volume of 3 μl on urine volume, FE*_Na_*, FEP*_Na_*, FEPP*_Na_* and FE_K_ in NP compared to age-matched LP offspring**.** Results are reported as mean±s.d. Data were analyzed using a two-way ANOVA test with *post-hoc* comparisons by Bonferroni's contrast test. The level of significance was set at *P<0.05. The study was controlled by the LV microinjection of 0.15 M NaCl.

**Fig. 6.**
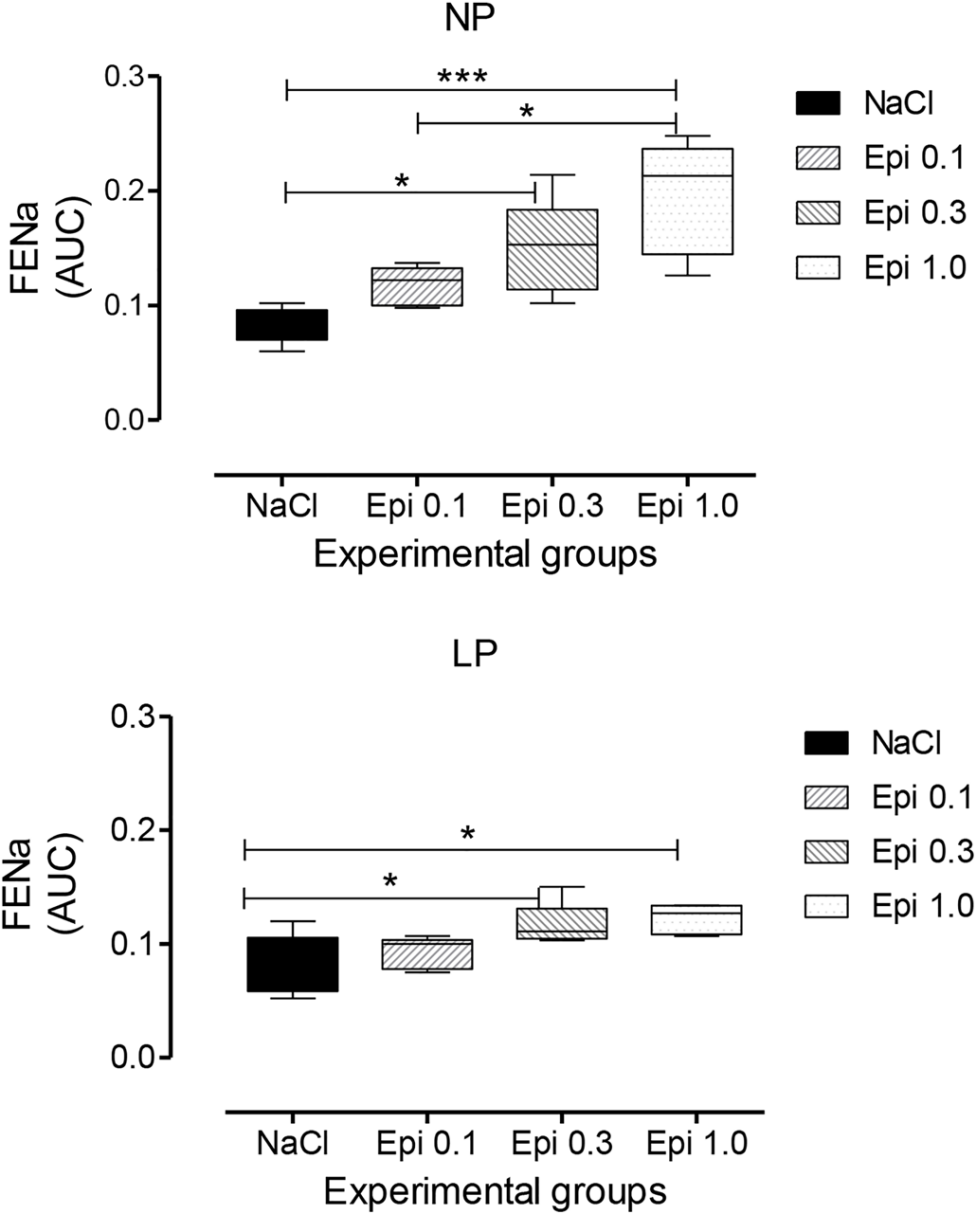
Dose-response curve to LV microinjection of agonists at different concentrations of Epi (0.1, 0.3 and 1.0 µmol) in a volume of 3 μl on FE*_Na_* expressed as mean±s.d. of the total area under the curve (AUC, %.120 min^-1^). Data were analyzed using a two-way ANOVA test with *post-hoc* comparisons by Bonferroni's contrast test. The level of significance was set at *P<0.05, **P<0.01, ***P<0.001. The study was controlled by the LV microinjection of 0.15 M NaCl.

**Fig. 7.**
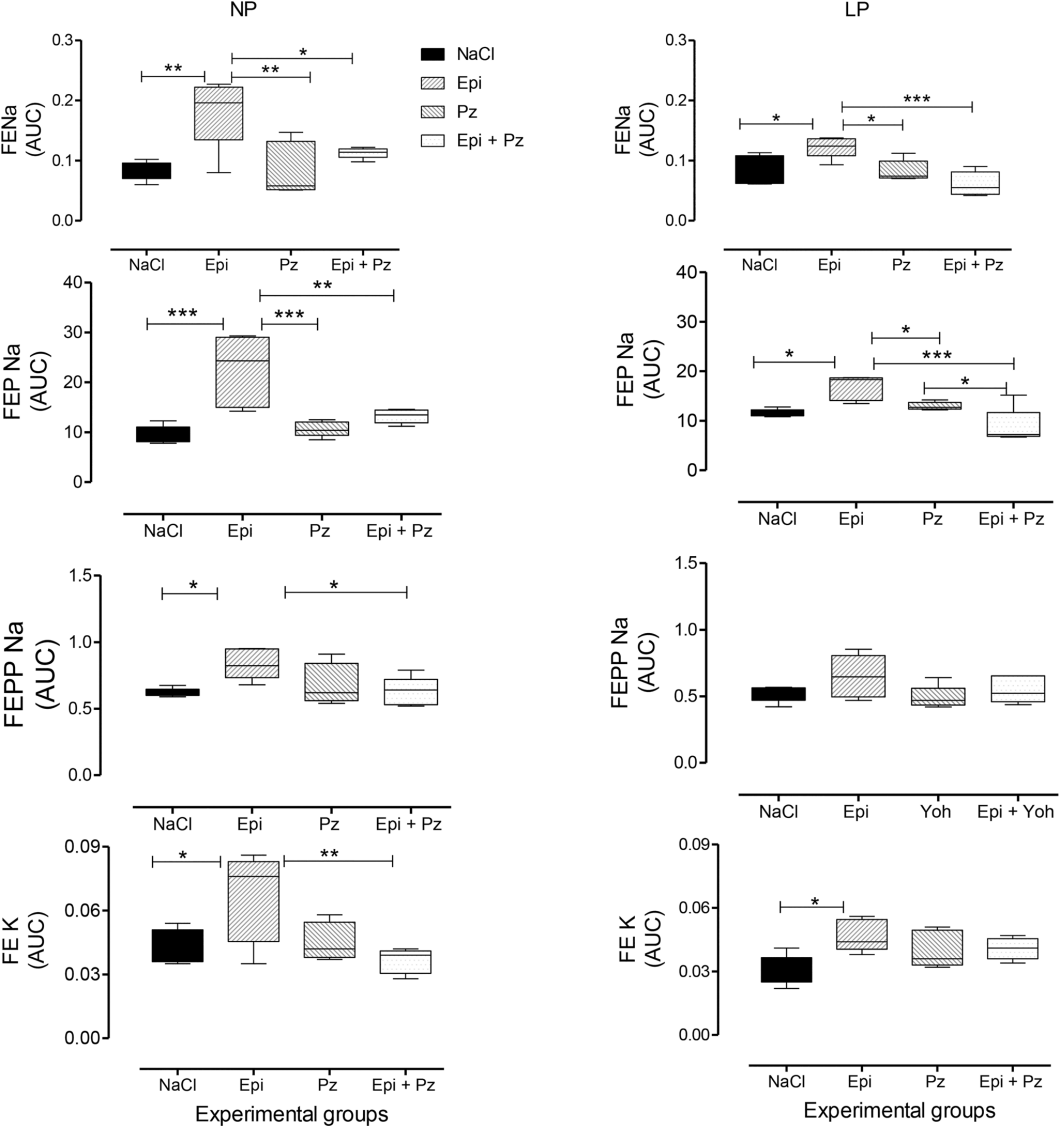
Effect of the LV microinjection of agonist 0.3-µmol Epi and/or α1-adrenoceptor antagonist, 4-nmol prazosin (Pz) on FE*_Na_*, FEP*_Na_*, FEPP*_Na_* and FE_K_ in NP compared to age-matched LP offspring. Results are reported as mean±s.d. Data were analyzed using a two-way ANOVA test with *post-hoc* comparisons by Bonferroni's contrast test. *P<0.05, **P<0.01, ***P<0.001. The study was controlled by the LV microinjection of 0.15 M NaCl.

Blood pressure and renal function data (expressed as mean±s.d.) for 16-week-old NP (*n*=10 to 32 offspring for each control group) and LP (*n*=10 to 36 for each experimental group) offspring are summarized in Figs 3–7 and Table 1. As shown in Fig. 2D, tail systolic arterial pressure (in mmHg) was significantly higher in LP offspring compared with NP offspring between 8 and 16 weeks of age (*P*=0.0001). The changes in systolic blood pressure from 8 to 16 weeks of age were as follows; 8 weeks: LP, 136.2±4.04 mmHg versus NP, 130.7±2.43 mmHg, *P*=0.0001; 16 weeks: LP, 156.1±6.25 mmHg versus NP, 134.4±4.04 mmHg, *P*=0.0001 (Fig. 2D). This study also revealed that in NP offspring there was a rapid, transient but considerable, blood pressure decrease after lateral ventricle (LV) microinjection of 0.3 µmol Epi (Fig. 4A,B; NP, *n*=9, *P*=0.0001) an effect which, in turn, was significantly attenuated by α1-adrenoceptor antagonists (4 nmol prazosin, *n*=10; *P*=0.2596) (Fig. 4A) and was unchanged by the α2-receptor antagonist (NP: 4 nmol yohimbine, *n*=10; *P*=0.1395) (Fig. 4B). No blood pressure changes, to LV microinjection of 0.3 µmol Epi or Epi+α1-adrenoceptor antagonists (*P*=0.0629 and *P*=0.3051, respectively), were observed in 16-week-old LP offspring. However, LV microinjection of 0.3 µmol Epi+α2-adrenoceptor antagonists caused a significant reduction in blood pressure in LP offspring (*P*=0.0007) (Fig. 4B).

### Renal function data-dose-response curve for Epi-induced sodium excretion response

**Fig. 8.**
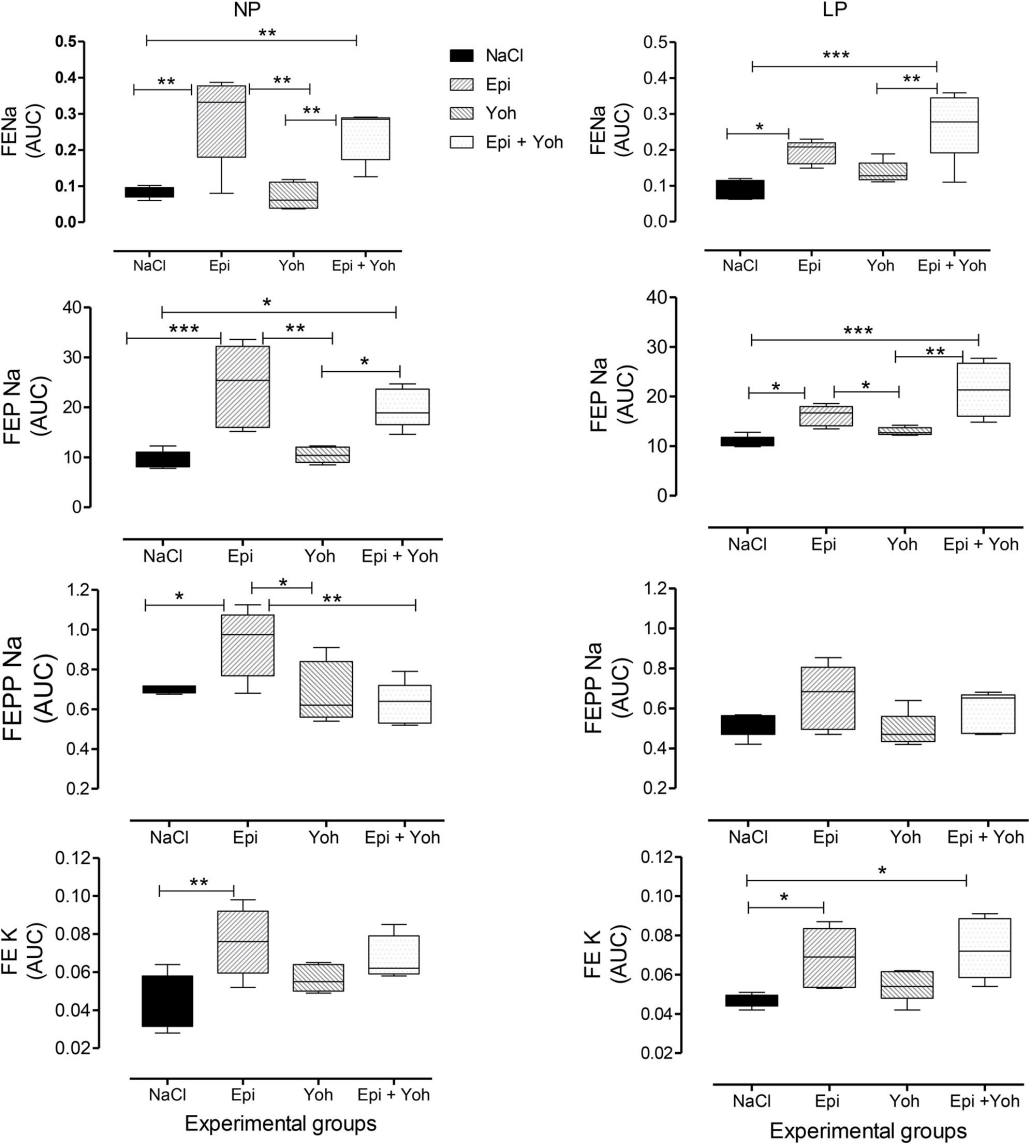
Effect of the LV microinjection of agonist 0.3 µmol Epi and/or their α2-adrenoceptor antagonist, 4 nmol yohimbine (Yoh), on FE*_Na_*, FEP*_Na_*, FEPP*_Na_* and FEK in NP compared to age-matched LP offspring. Results are reported as mean±s.d. Data were analyzed using a two-way ANOVA test with *post-hoc* comparisons by Bonferroni's contrast test. The level of significance was set at *P<0.05, **P<0.01, ***P<0.001. The study was controlled by the LV microinjection of 0.15 M NaCl.

Renal function in 16-week-old NP and LP offspring is summarized in Fig. 2. No changes in plasma sodium, lithium or potassium levels were observed in any of the experimental groups (Table 1). Also, the urinary flow rates (*P*=0.0782) and the glomerular filtration rate (*P*=0.1959), estimated by *C_Cr_* (creatinine clearance), did not significantly differ between the NP and LP offspring (Fig. 4A,D, respectively). Fractional urinary sodium excretion (FE*_Na_*, Fig. 4B) in 16-week old LP rats was significantly reduced in LP offspring relative to age-matched NP rats (16-week-old LP, 0.089±0.006% versus NP, 0.199±0.028%; *P*=0.006). The decreased FE*_Na_* in LP rats was accompanied by a significant reduction in fractional post-proximal sodium excretion (FEPP*_Na_*) (16-week-old LP, 0.324±0.031% versus NP, 0.589±0.07%; *P*=0.0013) and FEK (16-week-old LP, 0.046±0.006% versus NP, 0.0973±0.008%; *P*=0.0002), but not by fractional proximal sodium excretion (FEP*_Na_)* when compared with age-matched NP control rats (Fig 4C,E,F). Lateral ventricular (LV) microinjections, in a dose-dependent fashion of 0.1, 0.3 and 1.0 µmol Epi diluted in 3 μl volume promoted an increase in urinary sodium and potassium excretion over 120 min in 16-week-old NP; this effect was significantly (*P*=0.0001) attenuated in age-matched LP offspring (Figs 5 and 6). After dose-response experiments, a dose of 0.3 µmol Epi was selected as adequate for the rest of the study and the results showed as the total area under the curve (AUC, %.120 min^–1^). The effect of LV 0.3 µmol Epi on increasing renal fractional sodium excretion was significantly higher for NP than in LP offspring (*P*≤0.001) (Fig. 6). As depicted in Figs 6 and 7, a consistent increase of FE_*Na*_ among NP offspring was accompanied by significant enhancement of proximal (from basal 38.3±9.5 to 73.3±21.2%, *P*=0.0002) and post-proximal (from basal 38.3±9.5 to 73.3±21.2%, *P*=0.0245) sodium excretion. For the LP group, a smaller, but still significant, increase in proximal (from 42.9±8.3 to 54.7±7.6%) but not in post-proximal (from 2.36±0.38 to 5.12±0.62%) sodium excretion was observed (*P*=0.002 and *P*=0.001, respectively) (Fig. 5). The increase occurred in association with unchanged *C_Cr_*. The effects of LV antagonist adrenoceptor administration on urinary sodium excretion was also studied for both offspring groups bearing implanted cannulas. The study revealed the participation of LV α1 and α2-adrenergic receptors in the regulation of renal sodium and potassium excretion. The increased natriuresis and kaliuresis response to 0.3 μmol Epi microinjection into LV observed in 16-week-old NP rats, was significantly attenuated by previous local injection of an α1-adrenergic antagonist (4 nmol prazosin) (*P*=0.001) (Fig. 7). Additionally, that renal sodium excretion response was significantly blunted in 16-wk-old LP offspring relative to NP age-matched rats. Conversely, in NP rats, the current findings support the observation that LV pre-injection of 4-nmol yohimbine, an α2-adrenergic antagonist, synergically potentiates the action of 0.3 µmol Epi LV administration (Fig. 8) on renal sodium excretion in LP offspring. Note that prazosin (Fig. 7) significantly inhibited fractional sodium excretion whereas yohimbine (Fig. 8) enhanced fractional sodium excretion. Surprisingly, the LV yohimbine pretreatment normalized urinary sodium excretion by LP compared with age-matched NP offspring at 16 weeks of age. (*P*≤0.001, Fig. 8).

## DISCUSSION

The interaction between environmental and genetic factors interfere in ontogenic development, leading to morphofunctional disorders in tissues and organs in adulthood. Gestational protein restriction is followed by low birthweight in rats which in turn, leads to gender-related changes in blood pressure, kidney function, glucose metabolism and anxiety-like behaviors in male compared to female offspring. Sex hormones contribute to a sexual phenotype dimorphism in the fetal programming model of adult disease by modulating regulatory pathways critical in the long-term control of neural, cardiovascular and metabolic functions (Ashton, 2000; Barker, 1998; Mesquita et al., 2010a,b; Dasinger et al., 2016; Vaccari et al., 2015; Sene et al., 2013; Custódio et al., 2017; Menegon et al., 2008; Kwong et al., 2000; Ozaki et al., 2001; Gillette et al., 2017; Torres et al., 2018). Thus, this study was conducted only in male rats to ward off interference from gender differences. The current study confirms a reduced birth weight of rats whose mothers were fed a gestational restricted-protein diet compared to an NP intake (Mesquita et al., 2010a,b; Lopes et al., 2013). However, beyond the fourth week of age, body mass in both groups was the same, a phenomenon known as catch-up growth. This effect was associated with a significant enhancement in arterial blood pressure in the LP group. The present investigation also confirmed a pronounced decrease in fractional urinary sodium excretion in maternal protein-restricted offspring (Fig. 3). The decreased FE_*Na*_ observed in LP offspring compared with the age-matched NP group was accompanied by reduced post-proximal tubule sodium rejection, although the creatinine clearance was unchanged and sodium was usually filtered (3,4,41). The decreased renal potassium excretion verified in LP offspring suggests that tubular sodium reabsorption in LP offspring occurs before the distal nephron segment.

The precise mechanism underlying the chronic arterial hypertension in offspring induced by maternal LP has not been identified. Arterial pressure is thought to be controlled by the renal-mediated regulation of fluid and electrolytes. A prior report from our lab has shown higher renal and plasma catecholamine levels in LP offspring when compared to age-matched NP rats (Custódio et al., 2017). Also, as demonstrated in the previous study (Custódio et al., 2017), the bilateral renal denervation reduced kidney catecholamine concentrations in both NP and LP groups, though the decreased arterial blood pressure was observed only in growth-restricted offspring relative to renal denervated control rats. The enhanced urinary sodium excretion in kidney-denervated LP offspring suggests an indirect but close relationship between enhanced renal nerve activity and attenuated sodium excretion in the development of hypertension in LP offspring. The decreased FE_*Na*_^+^ in LP offspring may result from the interactions of a variety of mechanisms, such as renal arteriolar postglomerular vasoconstriction, renal sympathetic nervous system overexcitability and, by direct tubule transport effects; our previous study has demonstrated increased activity of the Na^+^/K^+^-ATPase pump in the basolateral membrane in LP rats (Mesquita et al., 2010a,b).

On the other hand, the adult kidney comprises several filtering units; in some species, total numbers of nephrons are determined before birth (Mesquita et al., 2010a; Hall et al., 2012). In rodents, the permanent metanephric kidney is very immature at birth and in rats about 20% of the total nephron number is present at birth. There is evidence that any insult, including maternal undernutrition offspring, alters the total number of nephrons and also causes late-onset hypertension (Mesquita et al., 2010a,b; Pinhal et al., 2013). However, in the present study, it does not seem that merely a reduced nephron number is responsible for the increased blood pressure, since we did not observe any significant difference between LP and NP glomerular filtration rate. Additionally, these findings are reiterated by data showing that impaired pelvic neurokinin expression associated with responsiveness of renal sensory receptors in 16-week-old LP offspring are conducive to excess renal reabsorption of sodium and development of hypertension in this programmed model (Custódio et al., 2017). Otherwise, investigators have demonstrated that administration of adrenergic agonists into different cerebral sites elicits a substantial increase in renal sodium excretion accompanied by decreasing arterial pressure (Gontijo et al., 1992; Pillar et al., 1977; Camargo et al., 1976; Saad et al., 1976; Lutaif et al., 2015). Thus, we hypothesized that enhanced blood pressure in maternal LP offspring could be associated, at least in part, with changes in renal neural control and reduced urinary sodium excretion that which may relate to imbalanced central adrenergic receptor modulation. Here we evaluated the effect of cerebro-LV administration of adrenergic agonists and/or antagonists on blood pressure and urinary sodium handling in 16-week-old LP offspring compared with appropriate age-matched NP controls in a concentration-dependent fashion. Of particular interest, we have confirmed results from different stimulation techniques (Gontijo et al., 1992; Pillar et al., 1977; Camargo et al., 1976; Saad et al., 1976; Lutaif et al., 2015; Kapusta et al., 1989; Koepke et al., 1988, 1987). This study reveals a rapid, transient but significant, blood pressure decrease after LV microinjection of Epi; this effect was, in turn, attenuated by α1-adrenoceptor antagonist and unchanged by α2-receptor antagonist ICV microinjections in NP rats. Surprisingly, only in LP offspring, the yohimbine LV microinjections, followed by Epi administration, cause a significant reduction in basal blood pressure, suggesting the centrally α1-adrenergic receptor participation in that pressure response.

Additionally, LV Epi microinjections, in a dose-dependent fashion, promoted an increase in urinary sodium and potassium excretion over 120 min in NP rats. Conversely, the natriuretic but not the pressure response to Epi microinjections into LV were significantly blunted in age-matched LP offspring. These findings confirm the participation of the CNS α1 and α2-adrenergic receptors in the regulation of renal sodium and potassium excretion. The increased natriuresis and kaliuresis response to LV Epi microinjections in NP rats were significantly attenuated by previous local injection of prazosin, an α1-adrenergic antagonist. Note that LV microinjections of prazosin inhibited fractional sodium excretion induced by Epi in NP and, to a lesser extent, in LP rats, whereas the current findings demonstrate that LV pre-injection of yohimbine, an α2-adrenergic antagonist, synergically potentiates and normalizes the action of ICV Epi administration on renal sodium excretion in age-matched LP offspring. Thus, this study confirms that Epi, when centrally microinjected in conscious rats, leads to a very predictable and reproducible natriuretic response accompanied by unchanged glomerular filtration rate and can be associated with an increased ion delivery from the proximal tubule, incompletely compensated by more distal nephron segments. This effect demonstrates diminished Epi graded-fashion responses with a rightward shift of the dose-response curve, providing evidence of downregulation of target organ responsiveness to LP cerebroventricular stimuli. Despite repeated demonstration of the natriuretic effect of central Epi administration to a variety of species, to the best of our knowledge, there has been no previous description of these effects among LP offspring.

However, the precise mechanism of this phenomenon remains unclear. Several possibilities could be considered to explain the natriuretic response in this study. First, the CNS directly affects renal sodium excretion via neural routes. Second, nephron hemodynamic changes are responsible for alteration of tubule electrolyte handling. Third, the natriuresis results from fluctuations in the level of presumable neural-borne factors which disrupt sodium and water transporters function in renal tubules. Fourth, the attenuated central response in LP relative age-matched NP offspring can supposedly be explained by a definite lack of control between centrally adrenergic and/or receptors activity that may blunt the peripheral kidney ion and salt excretion responses.

There is evidence of the importance of renal sympathetic nerve activity in the pathogenesis of experimental models of hypertension (DiBona, 2000; Johns et al., 2011; Boer et al., 2005). We previously demonstrated that the urinary sodium excretion response to central administration of insulin, angiotensin II, hypertonic saline and cholinergic and noradrenergic agonists were strikingly and similarly attenuated in different models of hypertensive rats when compared with age-matched normotensive controls (Custódio et al., 2017; Lutaif et al., 2015; Menegon et al., 2008; Guadagnini and Gontijo, 2006; Andersson et al., 1969). Thus, the significant reduced natriuretic response in LP compared to NP rats may reflect a hyperactive state in the peripheral sympathetic nervous system, including in the kidneys, at least in part caused by reduced sensory (afferent renal nerve activity) renal activity in LP offspring in adult life (Custódio et al., 2017; Boer et al., 2005; Oparil et al., 1987; Beierwaltes et al., 1982). This dysfunctional response in LP offspring could be essential to the development and maintenance of hypertension in LP offspring. In this way, it is well known that α2-adrenoceptors brainstem stimulation in the conscious rats causes a decrease in blood pressure and enhanced urinary sodium excretion. These effects are selectively mediated by downstream Gαi_2_, but not Gαi_1_, Gαi_3_, Gαo, or Gαs subunit GTP-binding regulatory protein signal transduction pathways (Kapusta et al., 1989, 2012; Koepke et al., 1988, 1987; Wainford and Kapusta, 2012). Studies revealed that the brain Gαi_2_ protein-mediated sympathetic inhibitory renal nerve-dependent path is of critical importance in the central neural mechanisms activated to maintain fluid and electrolyte homeostasis. The underlying mechanisms by which brain Gαi_2_-subunit protein-gated pathways induce α2-adrenoreceptor-evoked sodium and blood pressure control *in vivo* are unclear. Here, given the intimate association between fluid and electrolyte homeostasis and the long-term control of arterial pressure, we may speculate that downregulation of brain Gαi_2_ protein expression in LP offspring may lead to high kidney sympathetic drive, renal sodium retention and the development of renal nerve-dependent hypertension, effects partially disrupted by yohimbine LV microinjection. Our experiments furnished good evidence of the existence of a central adrenergic control mechanism consisting of α1 and α2 receptor signals, which work reciprocally on the regulation of blood pressure and renal sodium excretion. Speculatively, we may suppose that stimulation of CNS α2-adrenergic receptors by Gαi_2_ subunit GTP-binding regulatory protein, may prevent basal increased renal sympathetic overexcitability in conscious LP rats based on two main findings. First, the effect of LV administration of Epi on natriuresis is significantly attenuated in gestational protein-restricted offspring. Second, pretreatment with α2-adrenergic receptor antagonists reversed the impact of the LV Epi injection, which demonstrates that central α2-adrenergic receptors are involved in the diminished natriuresis observed for the LP lineage (Kapusta et al., 1989; Koepke et al., 1988, 1987). However, we cannot discount the possibility that LP neural synapses have more α2-adrenergic receptors than those of NP offspring. It is more likely that natriuresis is a result of reduced renal sympathetic nerve activity and a consequent decrease in renal tubular reabsorption of sodium, and a simultaneous consequent reduction in the blood pressure in LP offspring. Because adrenergic agonist or antagonists did not alter the glomerular filtration rate, changes in glomerular dynamics do not explain the natriuresis. Catecholamines administration into several CNS places in conscious rats increases urinary sodium excretion; the natriuresis is prevented by central α1-adrenergic receptor blockade and potentiated by α2-adrenergic receptor blockade (Gontijo et al., 1991, 1990; Kapusta et al., 1989; Koepke et al., 1988, 1987). Taking into account the current and previous studies, we may suggest an inhibitory effect of central α2-adrenergic receptors mediated, at least in part, by unbalanced downstream subunit GTP-binding regulatory protein on urinary sodium excretion and an excitatory effect of central α1-adrenoceptors.

In conclusion, our results suggest the striking participation of central adrenergic receptors in the renal pathogenesis of elevated blood pressure in LP offspring. Although the precise mechanism of the different natriuretic response of NP and LP rats is still uncertain, these results lead us to speculate that inappropriate neural adrenergic pathways may have significant effects on tubule sodium transport, resulting in the inability of the kidneys to control hydrosaline balance and, consequently, an increase in blood pressure.

## MATERIALS AND METHODS

### Animals and surgical procedures

The experiments were conducted as described in detail previously (Lutaif et al., 2015) on age-matched female and male rats of sibling-mated Wistar *HanUnib* rats (250–300 g) that were allowed free access to water and standard rodent chow (Nuvital, Curitiba, PR, Brazil). The Institutional Ethics Committee (CEUA/UNICAMP #2766/01) approved the experimental protocol, and the general guidelines established by the Brazilian College of Animal Experimentation were followed throughout the investigation. The environment and housing presented the right conditions for managing their health and wellbeing during the experimental procedure. Immediately after weaning at 3 weeks of age, animals were maintained under controlled temperature (25°C) and lighting conditions (07:00 h–19:00 h) with free access to tap water and standard laboratory rodent chow (Purina rat chow: Na^+^ content, 135±3 μEq/g; K^+^ content, 293±5 μEq/g), for 10 weeks before breeding. Day 1 of pregnancy was designated as the day in which the vaginal smear exhibited sperm. Dams were maintained on isocaloric rodent laboratory chow with standard protein content (NP; 17% protein) or low protein content (LP; 6% protein) diets, *ad libitum* intake, throughout the entire pregnancy. The NP and LP maternal food consumption was determined daily (subsequently normalized for body weight), and body mass of dams was recorded weekly in both groups. All maternal groups returned to the standard laboratory rodent chow after delivery. The male pups weaned for 3 weeks, and only one offspring from each litter was used for each experiment. The male offspring were also maintained with standard rodent chow under a controlled temperature (25°C), a 12 h light–dark cycle (07:00 h–19:00 h), and housed in cages with four rats until 16 weeks of age. The NP and LP offspring were handled weekly by a familiar person and followed until 16 weeks of age when physiological tests were performed. The litter number was determined, the anogenital distance was measured and male offspring were weighed at birth and weekly after that. Male NP and LP offspring (from 180–250 g) were chronically instrumented with a cerebral right LV guide cannula and kept under controlled temperature (25°C) and lighting conditions (07:00 h–19:00 h) in individual cages. Briefly, the animals were anesthetized with a mixture of ketamine (75 mg.kg-1 body mass, and xylazine 10 mg.kg-1 body mass), injected intraperitoneally (i.p.). Once the corneal and pedal reflexes were absent, a stainless steel cannula was stereotaxically implanted into the right LV, by use of techniques reported elsewhere, at pre-established coordinates: anteroposterior +0.2 mm from bregma; lateral +1.5 mm; and vertical −4.5 mm (Gontijo et al., 1992, 1991, 1990; Lutaif et al., 2015). After stereotaxic surgery, rats were allowed 1 week of recovery before testing for cannula patency and position. The position of the cannula was visually confirmed by infusion of Blue Evans 2% through the LV cannula at the end of the experiment (Fig. 1). Data from animals with incorrectly placed cannulas were excluded from statistical analysis.

### Renal function test

The effect of ICV adrenergic Epi microinjections on glomerular filtration and tubular sodium handling was assessed by creatinine and lithium clearance at 16 weeks of age (NP, *n*=10; LP, *n*=10), in conscious unrestrained male offspring. Briefly, 14 h before the renal test, 60 μmol LiCl/100g^−1^ body weight was given by gavage. The rats were subsequently housed in individual metabolic cages on overnight fast with free access to tap water. At 08:00 h, each animal received tap water by gavage (5% of body mass), followed by the same volume 1 h later. Immediately before experiments, the animals' (NP and LP) water supply was removed from the home cage. The indwelling obturator was replaced by a 30-gauge stainless steel injector at the end of PE-10 tubing connected to a 10-μl Hamilton syringe wholly filled with the test solution. 30 min after the second volume of water, Epi (*n*=9–10 for each dose, Sigma-Aldrich), or a similar amount of 0.15 M NaCl (vehicle, *n*=8) was microinjected into the LV in a volume of 3 μl, at different concentrations of Epi (0.1, 0.3 and 1.0 µmol) diluted in saline, of one NP or LP offspring from each litter. The drug was administered by a 10-μl Hamilton micro-syringe, and urine spontaneously voided over five 30 min-periods, one before (−30 min) and four after ICV drug administration (30–120 min), was collected into graduated centrifuge tubes and measured gravimetrically. To examine the effect of α-adrenergic receptor antagonist after LV administration of 0.3 µmol Epi, rats were randomly assigned to a specific experimental group and a selective α1-adrenoceptor antagonist (4-nmol prazosin, Pz; *n*=9, Sigma-Aldrich), α2-adrenoceptor antagonist (4-nmol Yohimbine, Yo; *n*=10, Sigma-Aldrich) or vehicle (0.15 M NaCl, *n*=8) was LV microinjected 30 min before the agonist in a volume of 1 μl. At the end of the experiment, blood samples were drawn by cardiac puncture from anesthetized rats, and urine and plasma samples were collected for analysis (Gontijo et al., 1991, 1990; Boer et al., 2005; Menegon et al., 2008).

### Blood pressure measurement

The systolic arterial pressure was measured in conscious and previously trained offspring at 8 to 16 weeks of age in NP (*n*=10 to 36) and LP (*n*=10 to 32) offspring. In a subgroup of animals, the systolic blood pressure (SBP) was measured 30 min before and after LV administration of 0.3 µmol Epi and/or receptor antagonists administration followed for two subsequent periods of 60 min, in conscious NP and LP offspring, employing an indirect tail-cuff method using an electrosphygmomanometer combined with a pneumatic pulse transducer and amplifier (IITC Life Science BpMonWin Monitor Version 1.33). This indirect approach enabled repeated measurements with close correlation (correlation coefficient=0.975), compared with direct intra-arterial recording (Mesquita et al., 2010a,b; Custódio et al., 2017; Boer et al., 2005; Menegon et al., 2008). The mean of three consecutive readings represented the blood pressure.

### Data presentation and statistical analysis

All numerical results are expressed as the mean±standard deviation (s.d.) or median and quartile deviation as appropriate. Plasma and urine sodium, potassium and lithium concentrations were measured by flame photometry (Micronal B262, São Paulo, Brazil). The integrated renal fractional sodium and potassium excretions after 0.3 µmol Epi LV microinjection, i.e. the total area under the curve (AUC, %.120 min^−1^) were calculated by the trapezoidal method. Plasma and urine sodium, potassium and lithium concentrations were measured by flame photometry (Micronal, B262, São Paulo, Brazil), while the creatinine concentrations and plasma osmolality were determined spectrophotometrically (Instruments Laboratory, Genesys V, USA) and by Wide-range Osmometer (Advanced Inst. Inc, MA, USA), respectively. After that, the animals were anesthetized with ketamine and xylazine injected intraperitoneally and euthanized by cardiac puncture; urine and plasma samples were stored for analysis. Creatinine clearance (*C_Cr_*) was assessed to estimate glomerular filtration rate, and lithium clearance (*C_Li_*) was used to determine proximal tubule output (Mesquita et al., 2010a,b; Custódio et al., 2017; Boer et al., 2005; Menegon et al., 2008). Fractional sodium excretion (FE*_Na_*) was calculated as *C_Na_*/*C_Cr_*×100, where *C*_Na_ is sodium clearance, and *C_Cr_* is creatinine clearance. Fractional proximal (FEP*_Na_*) and post-proximal (FEPP*_Na_*) sodium excretion were calculated as *C_Li_*/*C_Cr_*×100 and *C_Na_*/*C_Li_*×100, respectively. Data obtained over time were analyzed by the use of repeated measures two-way ANOVA. *Post-hoc* comparisons between selected means were performed with Bonferroni's contrast test when initial ANOVA indicated statistical differences between experimental groups. When relevant, comparisons involving only two means within or between groups were achieved by use of a Student's *t*-test. Statistical analysis was performed with GraphPad Prism 5.01 for Windows (1992-2007 GraphPad Software, Inc., La Jolla, CA, USA). The level of significance was set at *P*<0.05.
